# Genetic and morphological identification of a recurrent *Dicksonia* tree fern hybrid in New Zealand

**DOI:** 10.1371/journal.pone.0216903

**Published:** 2019-05-20

**Authors:** Lara D. Shepherd, Patrick J. Brownsey, Chris Stowe, Claire Newell, Leon R. Perrie

**Affiliations:** 1 Museum of New Zealand Te Papa Tongarewa, Wellington, New Zealand; 2 Urtica Ecology, Riverton, New Zealand; 3 Korowai Ecology, Springfield, New Zealand; Universita degli Studi di Napoli Federico II, ITALY

## Abstract

Hybridization is common in many ferns and has been a significant factor in fern evolution and speciation. However, hybrids are rare between the approximately 30 species of *Dicksonia* tree ferns world-wide, and none are well documented. In this study we examine the relationship of a newly-discovered *Dicksonia* tree fern from Whirinaki, New Zealand, which does not fit the current taxonomy of the three species currently recognized in New Zealand. Our microsatellite genotyping and ddRAD-seq data indicate these plants are F1 hybrids that have formed multiple times between *D*. *fibrosa* and *D*. *lanata* subsp. *lanata*. The Whirinaki plants have intermediate morphology between *D*. *fibrosa* and *D*. *lanata* subsp. *lanata* and their malformed spores are consistent with a hybrid origin. The Whirinaki plants–*Dicksonia fibrosa* × *D*. *lanata* subsp. *lanata*–are an example of hybridization between distantly related fern lineages, with the two parent species estimated to have diverged 55–25 mya. Our chloroplast sequencing indicates asymmetric chloroplast inheritance in the Whirinaki morphology with *D*. *lanata* subsp. *lanata* always contributing the chloroplast genome.

## Introduction

Tree ferns (Cyatheales) are often a locally dominant feature of wet temperate Southern Hemisphere forests. Within New Zealand’s forests tree ferns often represent a significant proportion of the forest community based on the number of individuals, biomass and basal area [1]. Two genera are represented in New Zealand’s tree ferns, *Cyathea* Sm. (Cyatheaceae) and *Dicksonia* L’Hér (Dicksoniceae) [2, 3]. The taxonomy of the New Zealand species in both of these genera is thought to be well understood, with no new species described since 1910 [2].

*Dicksonia* contains around 30 species that are found in Central and South America, Southeast Asia, eastern Australia, New Zealand and the Pacific [2,4]. Three indigenous *Dicksonia* species are recognized in New Zealand; all are endemic [2]. *Dicksonia fibrosa* Colenso (whekī-ponga) and *D*. *squarrosa* (G.Forst) Sw. (whekī) have trunked rhizomes up to 6m tall and 7m tall, respectively. Both are found throughout the North Island in lowland to montane forest and are largely confined to lowland and coastal areas in the South Island [2]. Two allopatric subspecies are recognized within *D*. *lanata* Colenso ex Hook. (stumpy tree fern; tūākura; tūōkura) [5]. *Dicksonia lanata* subsp. *hispida* (Colenso) Perrie & Brownsey has a trunked rhizome to 2m tall and is found in Northland and Auckland regions in the North Island. *Dicksonia lanata* subsp. *lanata* has a prostrate rhizome and is found mostly in montane habitats south of Auckland in the North Island and in coastal and lowland forest in north-western South Island.

A global chloroplast phylogeny of Dicksoniaceae [6] placed the New Zealand species into two of the three main clades of *Dicksonia*. *Dicksonia lanata* and *D*. *squarrosa* grouped in a clade with species from the Pacific Islands, whereas *D*. *fibrosa* formed another clade with species from Australia, Timor and South America. Molecular dating estimated these clades diverged 55–25 mya [6].

During a survey in 2015 of a remote area of Whirinaki Forest, eastern North Island, New Zealand, *Dicksonia* plants were discovered whose morphology did not fit with any of the existing species descriptions. They appeared to have fronds similar to *D*. *lanata* but had a stout trunk resembling that of *D*. *fibrosa*. Both *D*. *lanata* subsp. *lanata* and *D*. *fibrosa* grow in the immediate vicinity. Our subsequent searches located around 80 so-called Whirinaki plants over 3 km that could not be placed within the existing taxonomy. They were growing in sympatry with *D*. *lanata* subsp. *lanata*, *D*. *squarrosa* and *D*. *fibrosa*.

Using morphological and genetic (chloroplast sequences, microsatellite genotypes and ddRAD-Seq) data we examine whether the Whirinaki morphology is:

1. the Australian species *D*. *antarctica* Labill., which is sometimes confused with *D*. *fibrosa* [2], including sometimes claimed to be present in New Zealand;
2. an undescribed species;
3. a homoploid hybrid between *D*. *fibrosa* and *D*. *lanata* subsp. *lanata* that has formed once and has spread vegetatively (both *D*. *squarrosa* and *D*. *lanata* produce underground stolons from their rhizomes [2]);
4. a recurrent homoploid hybrid between *D*. *fibrosa* and *D*. *lanata* subsp. *lanata*;
5. a fertile allopolyploid between *D*. *fibrosa* and *D*. *lanata* subsp. *lanata*.

If the Whirinaki morphology is either *D*. *antarctica* (hypothesis 1) or an undescribed species (hypothesis 2) then, given its restricted distribution, it would likely be considered threatened under the New Zealand Threat Classification System [7]. Hypotheses (3), (4) and (5) all involve hybridization, which is rare in *Dicksonia*, with only unconfirmed reports of wild *Dicksonia* hybrids [4,8] and a single recorded hybridisation event in cultivation between *D*. *arborescens* L’Hér. from St Helena and *D*. *antarctica* [9].

## Materials and methods

### Sampling and DNA extraction

A total of 43 *Dicksonia* specimens were collected for this study (Table 1), with four to 14 individuals sampled for each of the New Zealand taxa, and one specimen of *D*. *antarctica*. Samples were collected under the Department of Conservation permit number CA-31615-OTH. Plants of the Whirinaki morphology were collected from two sites: c. 38° 48' S, 176° 37' E, and c. 38° 49' S, 176° 39' E. Fresh frond tissue was collected into silica gel. DNA was isolated from the dried tissue using a modified-CTAB extraction method (steps 1, 3–7 from Table 1 in [10]).

**Table 1:** Details of *Dicksonia* samples and genetic data generated in this study.

Species	Locality	WELT Voucher	Chloroplast GenBank Accession	Microsatellite genotypes (bp)			ddRAD-seq
				DicMic01	DicMic104	DicMic109	
*D*. *fibrosa*	Wellington	P028842	MK420406	316/316	114/114	131/131	Y
*D*. *fibrosa*	Whirinaki, site 1	P028843	MK420407	316/316	114/114	131/131	Y
*D*. *fibrosa*	Whirinaki, site 1	P028844	-	316/316	114/114	131/131	Y
*D*. *fibrosa*	Whirinaki, site 2	P028845	-	316/316	114/114	131/131	Y
*D*. *fibrosa*	Whirinaki, site 2	-	-	316/316	114/114	131/131	Y
*D*. *fibrosa*	Pohangina	P028846	-	316/316	114/114	131/131	-
*D*. *fibrosa*	Southland	P028847	-	316/316	114/114	131/131	Y
*D*. *fibrosa*	Southland	P028848	MK420408	316/316	114/114	131/131	-
*D*. *fibrosa*	Tokoroa	P024352	-	316/316	114/114	131/131	Y*
*D*. *lanata* subsp. *hispida*	Whangarei	P024320	MK420409	308/308	118/118	125/125	Y
*D*. *lanata* subsp. *hispida*	Whangarei	P024320	MK420414	312/310	118/118	125/125	Y
*D*. *lanata* subsp. *hispida*	Waipoua	P024332	MK420410	316/316	118/118	125/125	Y
*D*. *lanata* subsp. *hispida*	Waipoua	P024332	MK420411	325/310	118/118	125/125	Y
*D*. *lanata* subsp. *hispida*	Omahuta	P024325	MK420412	312/312	118/118	125/125	Y
*D*. *lanata* subsp. *hispida*	Omahuta	P024325	MK420413	316/308	118/118	125/125	Y
*D*. *lanata* subsp. *lanata*	Inangahua	P024340	MK420415	n/a	118/118	125/125	Y
*D*. *lanata* subsp. *lanata*	Coromandel	P024317	MK420416	n/a	118/118	125/125	Y*
*D*. *lanata* subsp. *lanata*	Matawai	P023528	MK420417	316/312	118/118	125/125	-
*D*. *lanata* subsp. *lanata*	Napier	P028849	MK420418	n/a	118/118	125/125	Y*
*D*. *lanata* subsp. *lanata*	Whirinaki, site 1	P028850	MK420419	n/a	118/118	125/125	Y
*D*. *lanata* subsp. *lanata*	Whirinaki, site 1	P028851	MK420420	312/312	118/118	125/125	Y*
*D*. *lanata* subsp. *lanata*	Whirinaki, site 2	P028852	MK420421	n/a	118/118	125/125	Y
*D*. *lanata* subsp. *lanata*	Whirinaki, site 2	-	-	316/306	118/118	125/125	Y
*D*. *squarrosa*	Pohangina	P024349	MK420422	n/a	121/121	125/125	Y
*D*. *squarrosa*	Aorangi	P028094	MK420423	n/a	118/118	125/125	Y
*D*. *squarrosa*	Whirinaki, site 1	P028853	-	n/a	118/118	125/125	Y
*D*. *squarrosa*	Whirinaki, site 1	P028854	-	n/a	121/121	125/125	Y
Whirinaki morphology	Whirinaki, site 1	P028855	MK420424	316/312	118/118	n/a	Y
Whirinaki morphology	Whirinaki, site 1	P028856	MK420425	316/316	118/118	131/125	Y
Whirinaki morphology	Whirinaki, site 1	P028857	MK420426	316/316	118/118	131/125	Y
Whirinaki morphology	Whirinaki, site 1	P028858	MK420427	316/316	118/118	131/125	Y
Whirinaki morphology	Whirinaki, site 1	P028859+	MK420428	316/316	118/118	131/125	Y
Whirinaki morphology	Whirinaki, site 1	P028860+	MK420429	316/316	118/118	131/125	Y
Whirinaki morphology	Whirinaki, site 1	P028861+	-	-	-	-	-
Whirinaki morphology	Whirinaki, site 1	P028862+	MK420430	316/310	118/118	131/125	Y
Whirinaki morphology	Whirinaki, site 1	P028863+	MK420431	316/310	118/118	131/125	Y
Whirinaki morphology	Whirinaki, site 1	P028864+	MK420432	316/306	118/118	131/125	Y
Whirinaki morphology	Whirinaki, site 2	P028865+	MK420433	316/312	118/118	131/125	Y
Whirinaki morphology	Whirinaki, site 2	P028866+	MK420434	316/316	118/118	131/125	Y
Whirinaki morphology	Whirinaki, site 2	-	MK420435	316/308	118/118	131/125	Y
Whirinaki morphology	Whirinaki, site 2	-	MK420436	316/316	118/118	131/125	Y
Whirinaki morphology	Whirinaki, site 2	-	MK420437	316/316	118/118	131/125	Y
*D*. *antarctica*	In cultivation	-	MK420438	316/316	113/113	131/131	Y#

+ included in examination of spore morphology

n/a sample failed to amplify.

* ddRAD-seq samples that were excluded from the initial assembly owing to low coverage.

# ddRAD-seq samples excluded from later analyses owing to low number of loci in the final assembly.

### Chloroplast sequencing and analyses

Six chloroplast loci were tested for variation in a subset of the New Zealand *Dicksonia* samples. The following loci and primers were trialled: *rps4*-*trnS* with T1 [11] and R1 [12], *trnG*-*trnR* with trnG1F and trnR22R [13], *psbA*-*trnH* with psbA [14] and trnH [15], *matK* with matKF_Dicksonia and matK_1R, *rpl16* with rpL16F_ferns and rpL16R_ferns and *trnL-trnF* with uv2 and uv4 [6].

PCR amplifications were performed in 12 μl reactions with 1× Mytaq mix (Bioline, Australia), 5 ρmol of each primer and 1 M betaine. PCR thermocycling conditions followed [16]. PCR products were visualized by agarose gel electrophoresis then purified by digestion with 0.5 U shrimp alkaline phosphatase (SAP, New England Biolabs, MA, USA) and 2.5 U exonuclease I (ExoI, New England Biolabs, MA, USA) at 37°C for 15 minutes, followed by inactivation of the enzymes at 80°C for 15 minutes. PCR fragments were sequenced in both directions with the ABI Prism Big Dye Terminator cycle sequencing kit version 3.1 on an ABI 3730 DNA sequencer (Massey University Genome Service, Palmerston North, New Zealand).

Sequence files were edited with Sequencher version 5.2 (Gene Codes Corp., Ann Arbor, MI, USA). The *trnL-trnF* region demonstrated the most variation and was sequenced for a total of 33 *Dicksonia* specimens, as described above. The *trnL-trnF* sequences contained only two unambiguous indels and these were aligned by eye. A median-joining network [17] for the *trnL-trnF* sequences was produced using PopArt [18] with indels (insertions and deletions) recoded as single events.

### Microsatellite analyses

Eleven microsatellite loci isolated from *Dicksonia sellowiana* Hook. [19] were tested in New Zealand *Dicksonia*. Primers to a further ten microsatellite loci developed from sequences obtained from a preliminary ddRAD-seq run on four *Dicksonia* (one each of *D*. *fibrosa*, *D*. *squarrosa*, *D*. *lanata* subsp. *lanata* and the Whirinaki morphology) were also screened. Primers were designed for these extra loci (S1 Table) using Primer3 [20].

An M13 tag (TGTAAAACGACGGCCAGT) was added to the 5’ end of the forward primer of each locus. Loci were amplified individually in 10 μL PCR reactions that contained 1 μL of diluted template DNA, 0.2 μM forward primer, 0.8 μM reverse primer, 0.8 μM M13 primer (labelled with FAM, NED, PET or HEX) and 1× MyTaq mix (Bioline). PCR thermocycling conditions were an initial denaturation of 94°C for 5 min followed by 35 cycles of 94°C for 20 s, 52°C for 20 s, and 72°C for 20 s and a final extension at 72°C for 15 min. For loci that amplified, genotyping was performed on an ABI 3730 DNA sequencer at the Massey Genome Service (Massey University, Palmerston North, New Zealand). Alleles were sized using the internal size standard GeneScan 500 LIZ (Applied Biosystems) and scored using Geneious version 10.2.6 (Biomatters Ltd., Auckland, New Zealand).

### ddRADseq library preparation and sequencing

Double-digest restriction site associated sequencing (ddRAD-Seq) libraries [21] were prepared for 39 individuals with 8 of these processed in duplicate as technical replicates. For each sample, 300 ng of DNA was digested with two restriction enzymes (AvaII and MspI, New England Biolabs Inc), following manufacturer’s instructions. These two enzymes were selected based on the recommendation of Yang et al [22], who found that this enzyme pair produced a high number of fragments for many plant species. Adaptors containing sample-specific barcodes and Illumina indices were ligated to each sample. Samples were pooled into three index pools and a size-selection performed on each pool for 300–500 bp fragments using excision from an agarose gel, followed by extraction with a Qiaquick gel extraction kit (Qiagen). Pooled size-selected samples were PCR-amplified to add Illumina indices using Phusion flash high fidelity PCR master mix (Thermo Scientific). Each pooled sample was purified and concentrated with a MinElute kit (Qiagen), quantified with a Qubit dsDNA HS (high sensitivity) assay kit (Thermo Fisher Scientific) and combined in equimolar amounts. A detailed protocol has been deposited in protocols.io (dx.doi.org/10.17504/protocols.io.zgyf3xw). The library was sequenced across a third of a lane using the Illumina HiSeq 2500 to generate 2 × 125 bp reads.

### ddRADseq assembly

Raw paired-end reads were demultiplexed with ipyrad v0.7.28 [23]. All Fastq sequence files are available from GenBank at the National Center for Biotechnology Information short-read archive database (accession number: PRJNA522301). Four samples had low numbers of reads and were removed prior to assembly. For the remaining samples, reads were demultiplexed, had their adaptors removed, and were merged and assembled into de novo loci using ipyrad (the params file we used in iyprad is provided in S1 Text). Reads were clustered at 90% similarity, with a minimum depth of coverage of six. Samples were treated as diploid allowing two alleles per site. Only loci present in at least 50% of the samples were retained.

A preliminary NeighborNet network (see below) was constructed from this initial assembly to confirm that the eight technical replicates clustered with their duplicate. The two duplicated sets of reads were then pooled and assembled as described above. The *D*. *antarctica* specimen produced a high number of raw reads but these were assembled into few loci compared with the other samples so this sample was excluded from the final dataset used for the analyses described below.

### ddRADseq data analyses

To examine whether there was conflicting phylogenetic signal in the ddRAD data, which may indicate a history of hybridization, a network was constructed with the NeighborNet algorithm [24], implemented in SplitsTree4 v4.14.6 [25] using uncorrected p-distances and the equal angle algorithm. Support with assessed with 1000 bootstrap replicates.

We used STRUCTURE v2.3.4 [26, 27] to examine genetic structuring without a priori inferences. A single snp was randomly selected from each ddRAD locus and the number of genetic clusters (K) was set between 1 and 5, with 10 permutations for each. We used the admixture model with correlated allele frequencies and ran STRUCTURE with a burn-in of 100,000 generations followed by 500,000 Markov Chain Monte Carlo (MCMC) iterations. The optimal number of genetic clusters (*K*) was determined by calculating the *ΔK* statistic [28] in STRUCTURE HARVESTER web v.0.6.94 [29]. We also examined all clustering results that warranted biological interpretation, following [30]. CLUMPP v.1.1.2 [31] was used to average iterative runs of *K* and the results visualized graphically with STRUCTURE PLOT [32].

### Morphology

The morphology of the Whirinaki plants was compared to *D*. *fibrosa* and *D*. *lanata*, based on published descriptions [2, 5]. The spores of 10 plants with the Whirinaki morphology were examined with a compound microscope. Spores were also examined from plants of *D*. *fibrosa* and *D*. *lanata* subsp. *lanata*.

## Results

### Chloroplast sequencing

**Fig 1:**
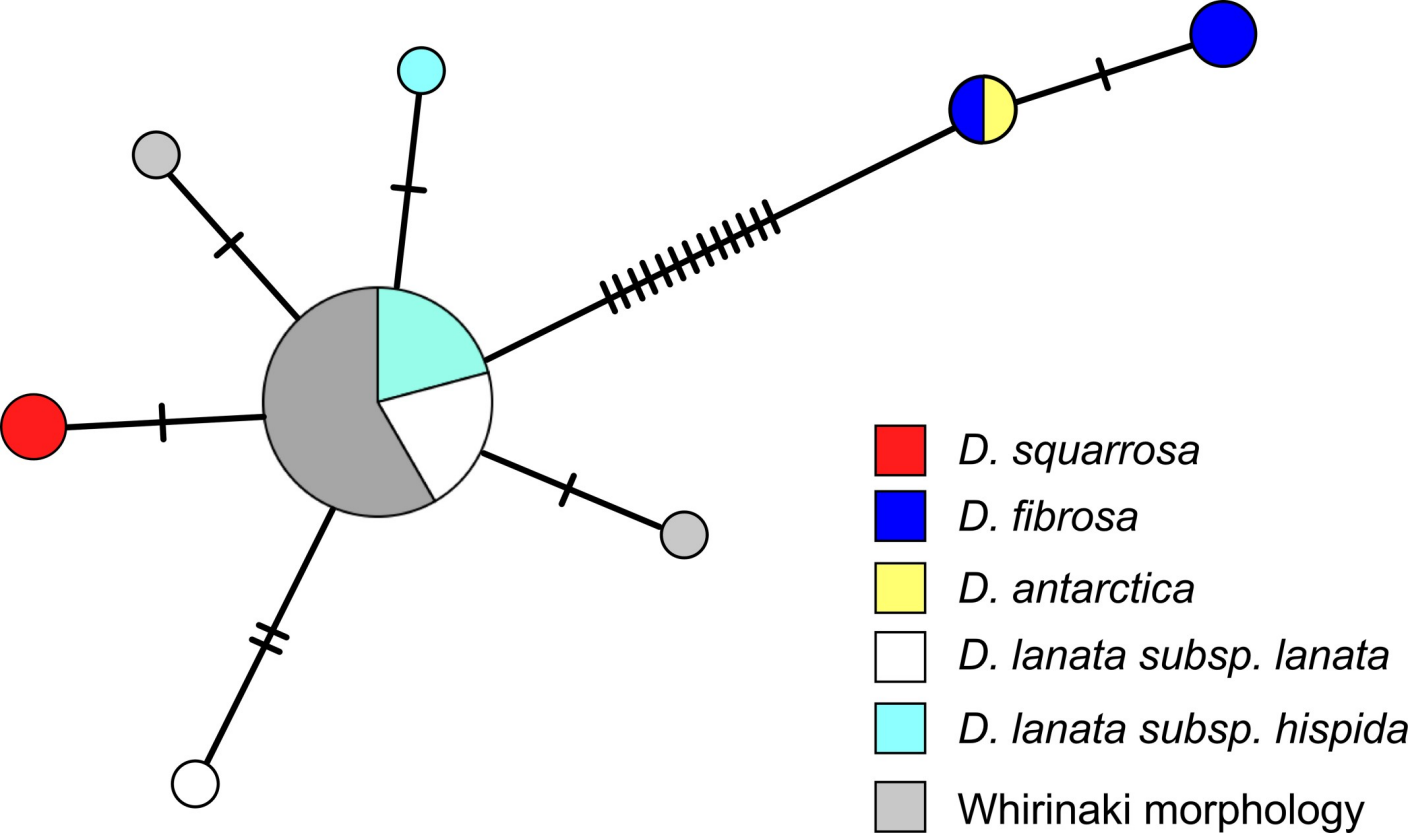
Median-joining haplotype network for the chloroplast *trnL-trnF* sequences in *Dicksonia*. The size of each circle is proportional to haplotype frequency. Hatch marks represent additional mutational steps separating haplotypes.

The *trnL-trnF* alignment was 972 bp in length and contained 17 substitutions and two unambiguous indel events. The relationships between the *trnL-trnF* sequences, with the indels coded, are shown in the median-joining network (Fig 1). The most common haplotype detected was shared by most of the *D*. *lanata* subsp. *lanata*, *D*. *lanata* subsp. *hispida* and Whirinaki morphology samples. Two additional haplotypes were found in samples with the Whirinaki morphology; each differed from the most common *D*. *lanata* haplotype by a single mutational change. Two haplotypes were restricted to *D*. *lanata*, one in each subspecies, and they differed from the common *D*. *lanata* haplotype by one or two substitutions. The two *D*. *squarrosa* samples had a haplotype that differed from the most common *D*. *lanata* haplotype by one mutation (a 10 bp insertion). The haplotypes detected in *D*. *antarctica* and *D*. *fibrosa* differed from the *D*. *lanata* haplotypes by a 6 bp insertion plus at least 12 substitutions. The *D*. *antarctica trnL-trnF* sequence was identical to one of the *D*. *fibrosa* sequences with the other two *D*. *fibrosa* sharing a haplotype that differed by one substitution.

### Microsatellites

Three microsatellite loci amplified and were variable within and/or between New Zealand *Dicksonia* species (Table 1). Neither *D*. *fibrosa* (n = 6) nor *D*. *antarctica* (n = 1) showed any variation at the three loci. *Dicksonia lanata* (n = 14) only varied at the DicMic01 locus, with six alleles detected. A number of *D*. *lanata* subsp. *lanata* samples failed to amplify at this locus suggesting that null alleles may be present in this taxon. *Dicksonia squarrosa* (n = 4) was invariant at the DicMic109 locus, had two alleles at the DicMic104 locus and failed to amplify at the DicMic01 locus. At locus DicMic109 all the individuals of the Whirinaki morphology (n = 14) were heterozygous for the 131 bp and 125 bp alleles. The 131 bp allele was the only allele detected at this locus in *D*. *fibrosa* and *D*. *antarctica* and the 125 bp allele was the only allele found in *D*. *lanata* and *D*. *squarrosa*. At locus DicMic104 the Whirinaki variant was fixed for a 118 bp allele, with this allele also fixed in *D*. *lanata* and also observed in *D*. *squarrosa*. At this locus *D*. *fibrosa* and *D*. *antarctica* only exhibited alleles 114 bp and 113 bp in size, respectively. At the third microsatellite locus, DicMic01, the Whirinaki variant displayed five alleles, all of which were also found in *D*. *lanata*. Every Whirinaki individual had at least one allele 316 bp in length and this allele was fixed in *D*. *fibrosa* and *D*. *antarctica*. *Dicksonia squarrosa* failed to amplify at this locus. Five different genotypes were found at this locus in the Whirinaki morphology.

### ddRAD-Seq

Illumina sequencing of *Dicksonia* ddRAD libraries for all 38 individuals plus 8 duplicates resulted in 303.48M reads after initial quality filtering. Excluding the four low coverage samples, 668K to 7.9M reads per individual were obtained (mean±SD = 3.43M±2.10M reads per sample) (Table 2).

**Table 2:** Summary of ddRAD-Seq data assembly following duplicate merging.

Species	Sample identifier	WELT Voucher	Number of raw reads	Clusters	Loci assembled	Average depth*	Heterozygosity	Sequencing error rate estimate
*D*. *lanata* subsp. *lanata*	3244	P024340	724578	319277	153	11.7	0.035919	0.011946
*D*. *lanata* subsp. *lanata*	7749	-	2556776	1444484	713	11.29	0.025572	0.007517
*D*. *lanata* subsp. *lanata*	7479	P028850	4716626	1149467	486	11.3	0.025825	0.008591
*D*. *lanata* subsp. *lanata*	7744	P028852	8964948	2449512	978	12.34	0.020612	0.005846
*D*. *lanata* subsp. *hispida*	7757_1	P024332	1189325	474517	213	11.88	0.035449	0.011587
*D*. *lanata* subsp. *hispida*	7757_2	P024332	1251222	573250	220	11.61	0.032657	0.010344
*D*. *lanata* subsp. *hispida*	7758_1	P024325	1430061	489037	321	12.42	0.03232	0.01019
*D*. *lanata* subsp. *hispida*	7758_3	P024325	1252782	516694	242	12.47	0.036091	0.010968
*D*. *lanata* subsp. *hispida*	7759	P024320	1378745	479375	507	13.22	0.025716	0.007436
Whirinaki morphology	7734	P028860	3336906	1199863	670	11.38	0.028035	0.008629
Whirinaki morphology	7738	P028862	2230355	943222	516	11.79	0.033381	0.010843
Whirinaki morphology	7481	P028858	7076904	1480722	890	12.19	0.019975	0.005847
Whirinaki morphology	7741	P028863	2024297	972612	537	11.93	0.027466	0.009158
Whirinaki morphology	7743	P028864	6003172	2031966	1108	11.48	0.026249	0.008476
Whirinaki morphology	7480	P028857	1540854	477947	698	13.03	0.029365	0.008805
Whirinaki morphology	7478	P028856	5418535	1242132	1168	12.7	0.023254	0.007184
Whirinaki morphology	7746	P028865	2426558	1025272	692	12.49	0.029059	0.009629
Whirinaki morphology	7747	P028866	2799126	1093751	805	11.77	0.0254	0.008994
Whirinaki morphology	7476	P028855	7918804	3178266	1110	10.88	0.021951	0.006344
Whirinaki morphology	7729	P028859	2204883	967748	573	11.3	0.023833	0.007026
Whirinaki morphology	7751A	-	5724685	2496619	841	10.9	0.024114	0.007181
Whirinaki morphology	7751C	-	4595418	1817517	947	11.33	0.026875	0.009065
Whirinaki morphology	7751E	-	5165004	1611692	1067	11.61	0.025422	0.008668
*D*. *fibrosa*	CS17616	P028847	8821385	479375	1061	11.64	0.018986	0.00606
*D*. *fibrosa*	CS27616	P028848	8821385	2990320	232	11.9	0.021972	0.005911
*D*. *fibrosa*	7750	-	6211962	710821	1085	12.19	0.017567	0.006465
*D*. *fibrosa*	7519	P028842	3813302	1737956	844	11.87	0.021111	0.007571
*D*. *fibrosa*	7477	P028843	5654226	1133382	1088	12.63	0.016313	0.00616
*D*. *fibrosa*	7730	P028844	6270854	1417517	1060	12.75	0.016761	0.00649
*D*. *fibrosa*	7745	P028845	11328979	1471468	1133	14.03	0.014535	0.005369
*D*. *squarrosa*	3413	P024349	1514166	2435906	277	11.92	0.032241	0.009651
*D*. *squarrosa*	7502	P028094	6667110	491128	1167	12.04	0.021597	0.006074
*D*. *squarrosa*	7733	P028853	6875753	2207477	916	12.33	0.019101	0.005528
*D*. *squarrosa*	7739	P028854	3458185	1570744	755	11.65	0.021847	0.006386

* after excluding loci with depth <6

The preliminary NeighborNet including the duplicate samples and the *D*. *antarctica* sample is shown in S1 Fig. It demonstrates that the duplicates largely cluster together and that the *D*. *antarctica* sample is closely related to *D*. *fibrosa*. The duplicates were combined for subsequent analyses and the *D*. *antarctica* sample was excluded owing to low coverage.

**Fig 2:**
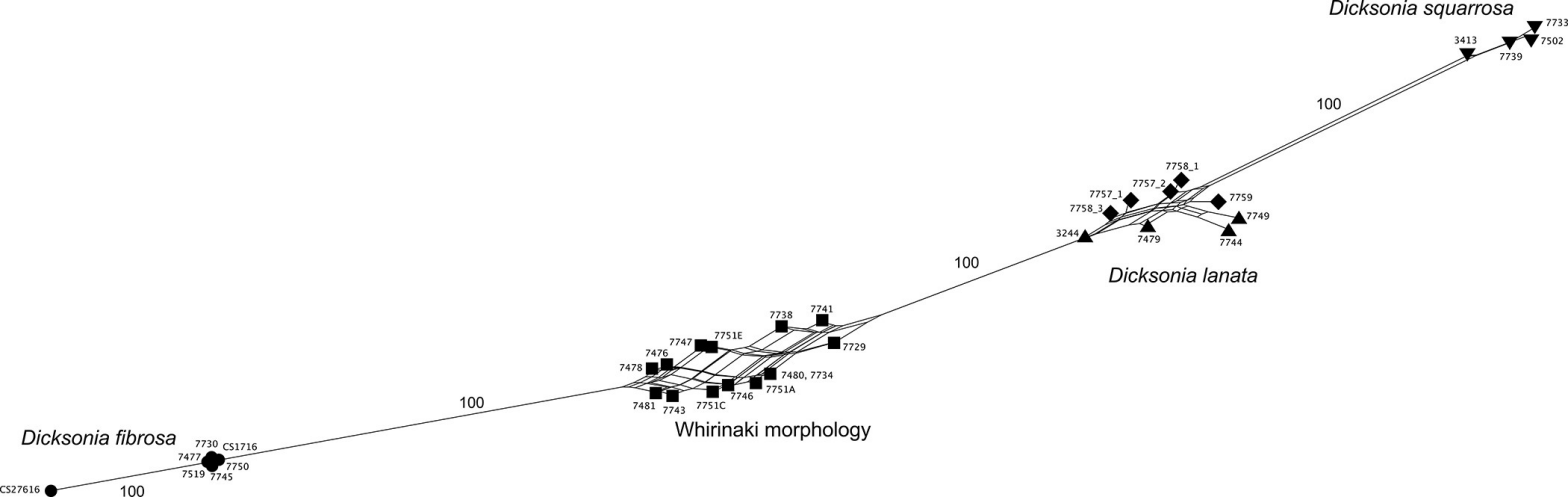
NeighborNet phylogenetic network derived from 1244 ddRAD-Seq loci. *Dicksonia lanata* subsp. *lanata* samples are represented by upward facing triangles and *D*. *lanata* subsp. *hispida* samples are shown as diamonds. Bootstrap support values are shown.

The final dataset comprised 1244 loci across 33 specimens. The NeighborNet analysis of this dataset clustered each species together with strong support (Fig 2). There was little variation within *D*. *fibrosa* and *D*. *squarrosa*. In contrast both *D*. *lanata* subsp. *lanata* and *D*. *lanata* subsp. *hispida* exhibited greater variation between individuals and while there was not strong evidence for the separation of these two subspecies, they were somewhat separated in the network.

Individuals of the Whirinaki morphology grouped together in an intermediate position between *D*. *fibrosa* and *D*. *lanata*.

**Fig 3:**
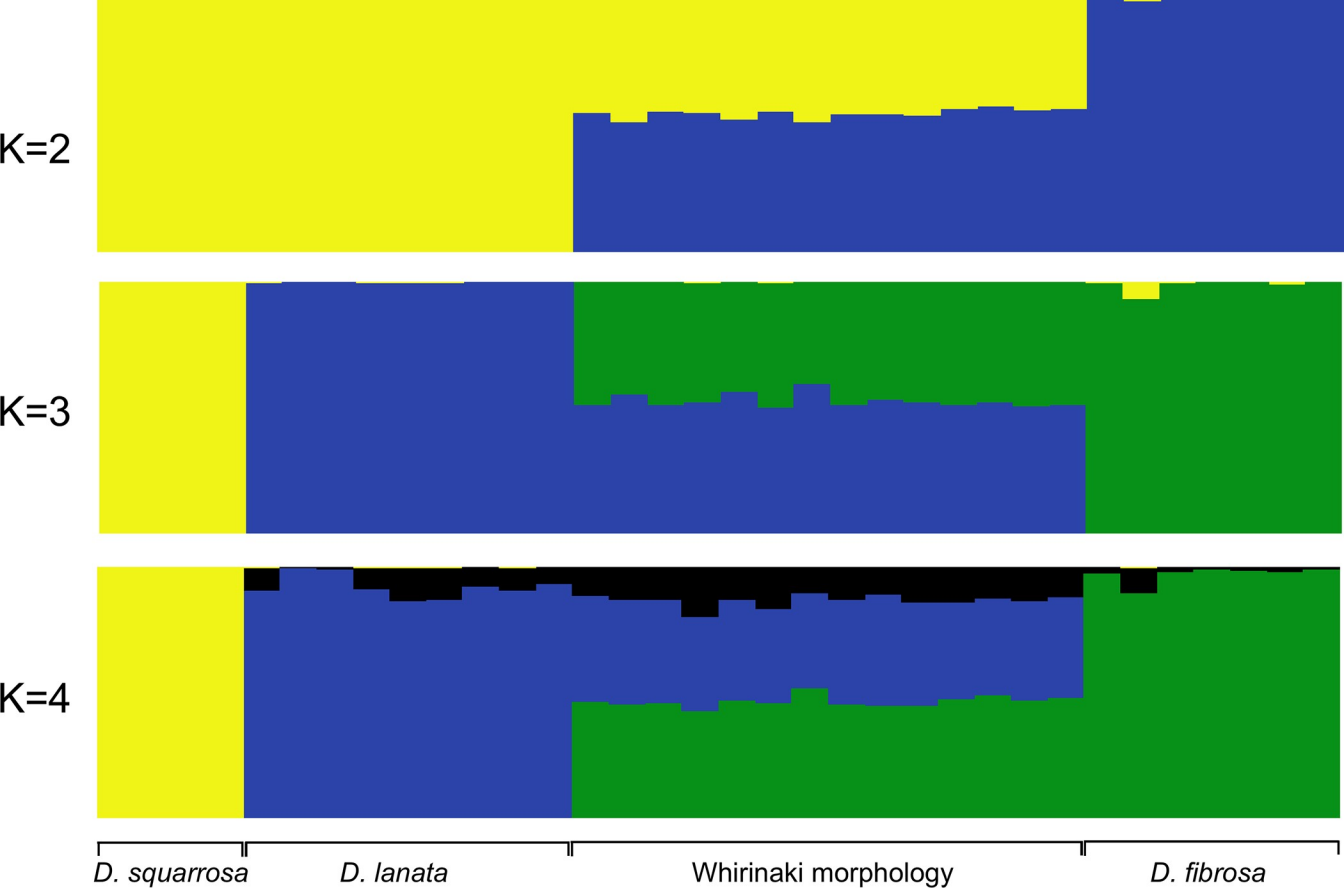
STRUCTURE plot of *Dicksonia* populations for K = 2 to K = 4 based on 1244 ddRAD-Seq loci.

For the Structure analyses *ΔK* indicated that the optimal K was 2 (S2 Fig; Fig 3). At K = 2 *D*. *lanata* and *D*. *squarrosa* were assigned to a single cluster and *D*. *fibrosa* was assigned to a second cluster. Individuals of the Whirinaki morphology were assigned equally to each of these two clusters. At K = 3 *D*. *squarrosa* was assigned with high probability to a third cluster while the Whirinaki morphology individuals were assigned equally between the *D*. *lanata* and *D*. *fibrosa* clusters. At K > 3 individuals were partitioned as for K = 3 but additional clusters were partitioned across all samples.

### Morphology

**Fig 4:**
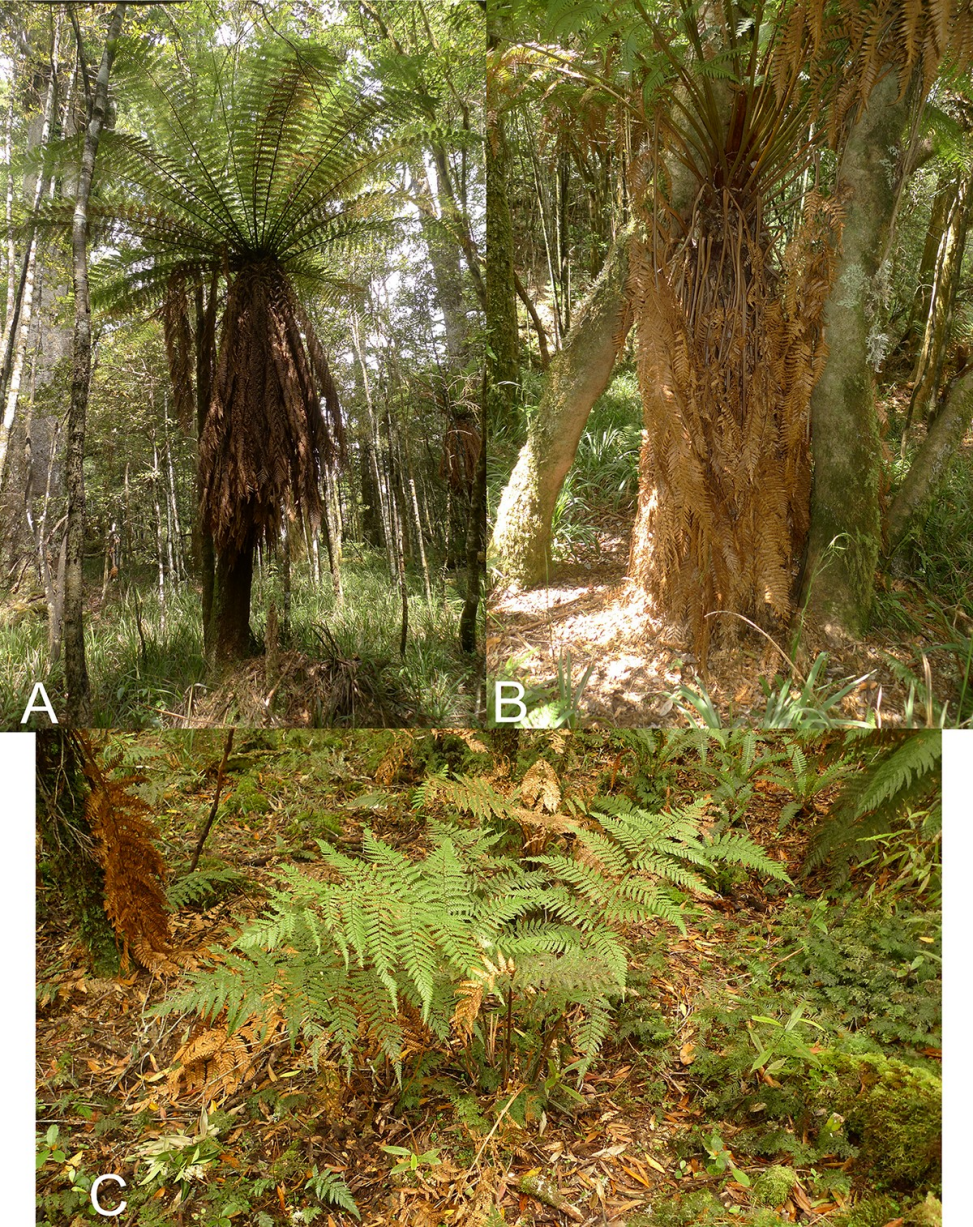
Field images of *Dicksonia*, Whirinaki Forest. (A) *Dicksonia fibrosa* showing the trunk and skirt of dead fronds. (B) *Dicksonia fibrosa* × *D*. *lanata* subsp. *lanata* with its trunk and less-developed skirt of dead fronds compared with *D*. *fibrosa*. The long stipes are also visible. (C) *Dicksonia lanata* subsp. *lanata* from Matawai with its lack of trunk.

**Fig 5:**
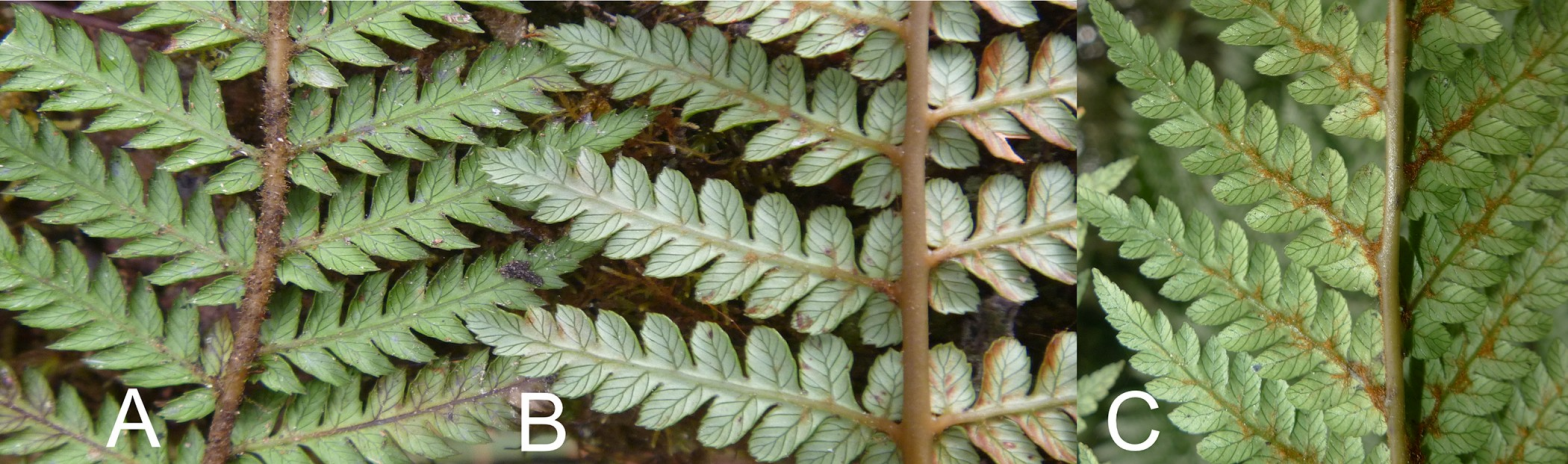
Field images of *Dicksonia* fronds, Whirinaki Forest. Frond undersides of (A) *Dicksonia fibrosa*, (B) *Dicksonia fibrosa* × *D*. *lanata* subsp. *lanata*, (C) *Dicksonia lanata* subsp. *lanata* showing woolly hairs.

About 80 individuals of the Whirinaki morphology were observed in the field in the Te Kohu area of Whirinaki Forest at an altitude of 800-920m above sea level. Plants with the Whirinaki morphology have a wide adventitious root-covered trunk similar to that of *D*. *fibrosa* but never exceeding 2m in height, whereas *D*. *fibrosa* is regularly taller (Table 3). *Dicksonia fibrosa* and the Whirinaki morphology also share skirts of dead fronds but the skirt is less well developed in the latter (Fig 4). The Whirinaki morphology is most easily distinguished from *D*. *fibrosa* by its longer stipes, which are usually orange-brown (only one of the 80 observed plants had green stipes), its wider fronds and the obtuse apices of its lamina segments (Table 3, Fig 5). Also, unlike *D*. *fibrosa*, the Whirinaki morphology has sparse brown woolly hairs up to 5mm long in the undersides of the rachis, pinna midribs and costae, especially in clusters in the coastae junctions. Similar hairs also occur in *D*. *lanata* subsp. *lanata* but the hairs are much denser than in the Whirinaki morphology.

**Table 3:** Morphological comparison of the Whirinaki morphology with *Dicksonia lanata* and *D*. *fibrosa*.

Character	Whirinaki morphology- *Dicksonia fibrosa* × *D*. *lanata* subsp. *lanata*	*D*. *fibrosa*	*D*. *lanata* subsp. *lanata*	*D*. *lanata* subsp. *hispida*
Trunk	Trunk covered in adventitious roots and up to 2m tall and skirted with dead fronds	Trunk covered in adventitious roots and up to 6m tall and skirted with dead fronds	Trunkless	Trunk up to 1.5m tall covered in stipe bases and adventitious roots and no skirt of dead fronds
Stipe length	38-75cm	5-50cm	23–100 cm	21-117cm
Stipe colour	Orange-brown or rarely green	Green adaxially, and green, pale or red-brown abaxially	Red-brown at base becoming yellow-brown or green distally	Red-brown at base becoming chestnut-brown, yellow brown or green distally
Lamina length	100–190 cm	95–280 cm	24–76 cm	42–113 cm
Lamina width	48–68 cm	21–60 cm	14–50 cm	20–70 cm
Tertiary pinnae lamina apex shape	Obtuse	Acute	Obtuse	Obtuse
Hairs of the undersides of the rachis, pinna midribs and costae	Sparse fine pale brown or chestnut brown hairs, woolly, curled or with occasional straight ones, up to 5 mm long, in clusters at costa junctions but less dense that in *D*. *lanata* subsp. *lanata*	Fine uniformly distributed colourless, pale brown or chestnut brown hairs up to 1 mm long	Fine pale brown or chestnut brown hairs, woolly, curled or with occasional straight ones, up to 5 mm long, in dense clusters at costa junctions	Fine uniformly distributed colourless or pale brown hairs, up to 1 mm long, straight or slightly curled, interspersed with red–brown, thicker, rigid, multicellular hairs up to 2.5 mm long

**Fig 6:**
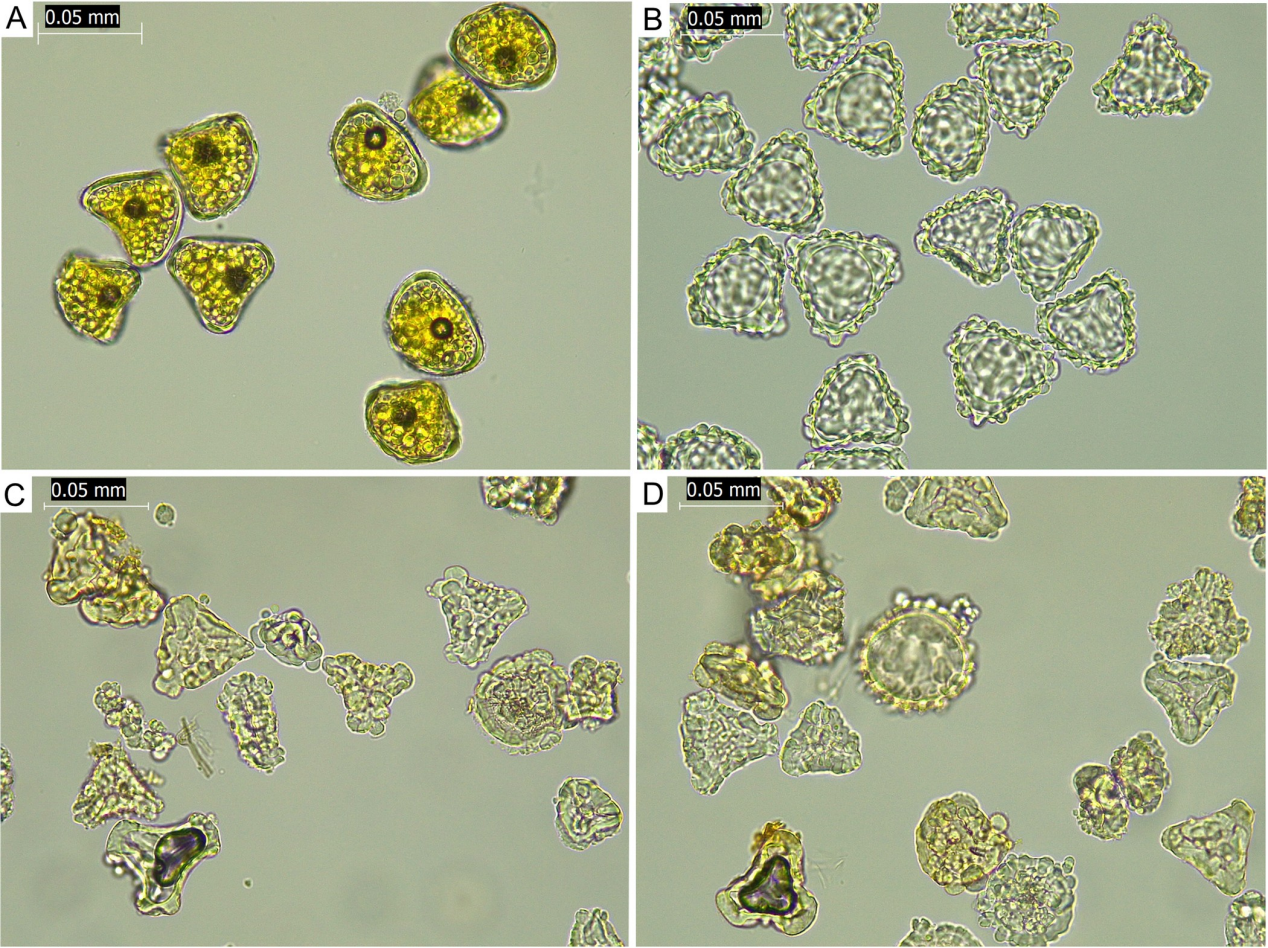
*Dicksonia* spore images. (A) *Dicksonia fibrosa*, (B) *Dicksonia lanata* subsp. *lanata*, (C) *Dicksonia fibrosa* × *D*. *lanata* subsp. *lanata*, (D) *Dicksonia fibrosa* × *D*. *lanata* subsp. *lanata*.

The spores examined from each of the ten accessions of the Whirinaki morphology were malformed and variable in size but generally smaller than those of *D*. *fibrosa* and *D*. *lanata* subsp. *lanata* (Fig 6). They also had no obvious exospore compared to *D*. *fibrosa* and *D*. *lanata* subsp. *lanata*.

## Discussion

### The origin of the Whirinaki morphology of *Dicksonia* involves hybridisation

Our genetic results indicate that the plants with the Whirinaki morphology are not *Dicksonia antarctica* (hypothesis 1) because they differ in their chloroplast sequences and do not cluster together in the NeighborNet of the ddRAD-Seq data. Instead our analyses indicate that the Whirinaki morphology plants are hybrids between *D*. *fibrosa* and *D*. *lanata*. The NeighborNet of the ddRAD-Seq data placed samples of the Whirinaki morphology at an intermediate position between these two species and the STRUCTURE analyses assigned them approximately equally to the clusters containing *D*. *fibrosa* and *D*. *lanata*. Two of the microsatellite loci (DicMic01, DicMic109) also provided support that the Whirinaki morphology plants are hybrids between *D*. *fibrosa* and *D*. *lanata*. Only the third microsatellite locus (DicMic104) did not show a pattern consistent with hybridization because *D*. *lanata* and the Whirinaki morphology were fixed for the same single allele and *D*. *fibrosa* was fixed for a different allele. None of the *Dicksonia* individuals genotyped were heterozygous at this locus and it is possible that this locus derives from a uniparentally-inherited organelle.

The two subspecies of *Dicksonia lanata* are only weakly separated in the ddRAD-Seq analysis, and not at all in the STRUCTURE analysis. This may reflect the make-up of the sample set, particularly the inclusion of hybrids. The AFLP analysis of a larger sample set by Lewis cited in [5] recovered strong support for separation of the two subspecies. This, together with morphological and geographic evidence, means we think there are strong grounds for maintaining them as separate taxa [2]. Furthermore, while the genetic results do not implicate one subspecies over the other in the hybridisation, only *D*. *lanata* subsp. *lanata* occurs in close proximity, and morphologically the Whirinaki plants show characters of both *D*. *lanata* subsp. *lanata* (cf. subsp. *hispida*) and *D*. *fibrosa* (Table 3).

The abnormal spores observed in the Whirinaki morphology indicate that these plants are infertile F1 hybrids and the genetic analyses largely also support this conclusion. Particularly informative was the DicMic109 microsatellite locus where all individuals of the Whirinaki morphology were heterozygous for the same two alleles, one of which was fixed in *D*. *lanata* and the other was fixed in *D*. *fibrosa*. This is the expected allele pattern for F1 hybrids (homozygotes, as well as heterozygotes, would be predicted for other hybrid classes such as F2 and backcrosses). Furthermore no more than two alleles were detected per locus at the microsatellite loci, as would be expected for diploid hybrids. The high level of variation observed in the Whirinaki morphology in the ddRAD NeighborNet and at microsatellite locus DicMic04 indicates that this form has not arisen from a single hybridization event (hypothesis 3) but from repeated crosses between genetically differentiated parent individuals (hypothesis 4). The three chloroplast haplotypes found in the Whirinaki morphology also supports recurrent hybridization.

### Asymmetric hybridization

The chloroplast haplotypes sequenced from the 15 hybrids were identical or very similar to the haplotypes detected from *D*. *lanata*, indicating that this species was the chloroplast donor. This 15:0 ratio is statistically different from the 1:1 expectation if *D*. *lanata* and *D*. *fibrosa* were equal contributors of chloroplasts to the hybrids (*χ*^2^ = 15, d.f. = 1, p = 0.0001). Asymmetric hybridization has been noted previously in ferns [33–36] but the drivers remain poorly understood [35]. Factors such as the relative abundance of the parental species’ gametophytes, differences between parent species’ sperm size and dispersability and sizes of the archegonial neck canal, differential success of embryos from reciprocal crosses and whether parent species exhibit an antheridiogen system may contribute to asymmetric hybridization [35].

### Hybridisation in *Dicksonia*

Our finding that the Whirinaki morphology comprises recurrent F1 hybrids was surprising for several reasons. Firstly, these *Dicksonia fibrosa* × *D*. *lanata* subsp. *lanata* plants are the first wild hybrids confirmed by genetics in the genus but they were locally common (although they represented only a small fraction of the tree fern community in the area). Despite being such a large plant, it is possible that *Dicksonia fibrosa* × *D*. *lanata* subsp. *lanata* plants occur elsewhere but have been overlooked. However, although the distributions of *D*. *fibrosa* and *D*. *lanata* broadly overlap over a wide area [2], the New Zealand National Vegetation Survey Databank suggests that the two species rarely grow in close proximity, which would limit their opportunities for hybridization. Of the 97555 permanent mainland plots in the New Zealand National Vegetation Survey Databank [37], *D*. *fibrosa* and *D*. *lanata* (either subspecies) are recorded from 1006 and 791 plots, respectively, but only occur together in 15 of these plots. Differences in ecological preferences may account for this lack of local overlap, with *D*. *lanata* subsp. *lanata* preferring hillsides and *D*. *fibrosa* more likely to be found on gully floors. The western Whirinaki area may be unusual in that the two species occur together over a large area, possibly because it is flatter, colder and drier forest than many other sites [38]. Examining other sites where *D*. *fibrosa* and *D*. *lanata* are sympatric may reveal further hybrids and provide insight into the environmental conditions that lead to hybridization between these two species.

Secondly, the hybridization has occurred between *Dicksonia* from chloroplast clades that have been estimated to have diverged 55–25 mya [6]. The Whirinaki morphology therefore provides another example of hybridization between deeply diverged fern lineages as has been found between *Cystopteris* and *Gymnocarpium* (~60 mya [39]) and *Asplenium* (45 mya [36]). *Dicksonia squarrosa* and *D*. *lanata*, which are widely sympatric and much more closely related to each other than either is to *D*. *fibrosa* [6], are not known to hybridise.

Lastly, although recurrently formed hybrids, *Dicksonia fibrosa* × *D*. *lanata* subsp. *lanata* exhibited remarkably conserved morphologies given the considerable differences between the morphologies of the parents.

Polyploidy is thought to be a significant process in the generation of fern diversity with an estimated 31% of fern speciation events accompanied by an increase in ploidy [40]. However, while many fern genera hybridize frequently and have highly duplicated genomes (e.g., *Asplenium* [41, 42], others, such as *Dicksonia*, hybridize only rarely, leading to limited opportunities for polyploidy. Understanding the situations where the barriers preventing hybridization break down in rarely hybridizing genera such as *Dicksonia* may provide insight into the dynamics of fern evolution and a contrast to genera with frequent reticulate evolution.

We are not making a nothospecies name because the New Zealand practice for naming fern hybrids is to use a hybrid binomial i.e., *Dicksonia fibrosa* × *D*. *lanata* subsp. *lanata*.

## Supporting information

S1 Fig NeighborNet for New Zealand *Dicksonia* based on 446 ddRAD-Seq loci. Duplicates are indicated by ‘dup’ after sequence name and were combined for subsequent analyses.
(TIF)

S2 Fig Results of implementing the Evanno method for detecting the number of K groups that best fit the *Dicksonia* ddRAD-Seq data. According to the ΔK, K = 2 represents the optimal structure partition in our dataset.
(PDF)

S1 Table Newly developed microsatellite primers for New Zealand *Dicksonia*. (DOCX)

S1 Text Ipyrad params file used for processing *Dicksonia* ddRAD-Seq data. (TXT)
