# Supplementary figures and images for: Genetic and morphological identification of a recurrent Dicksonia tree fern hybrid in New Zealand
Source: PLoS One. 2019 May 20;14(5):e0216903. doi: 10.1371/journal.pone.0216903 (PMC6527230; doi:10.1371/journal.pone.0216903)

$$\text{DeltaK} = \text{mean}(|L''(K)|) / \text{sd}(L(K))$$

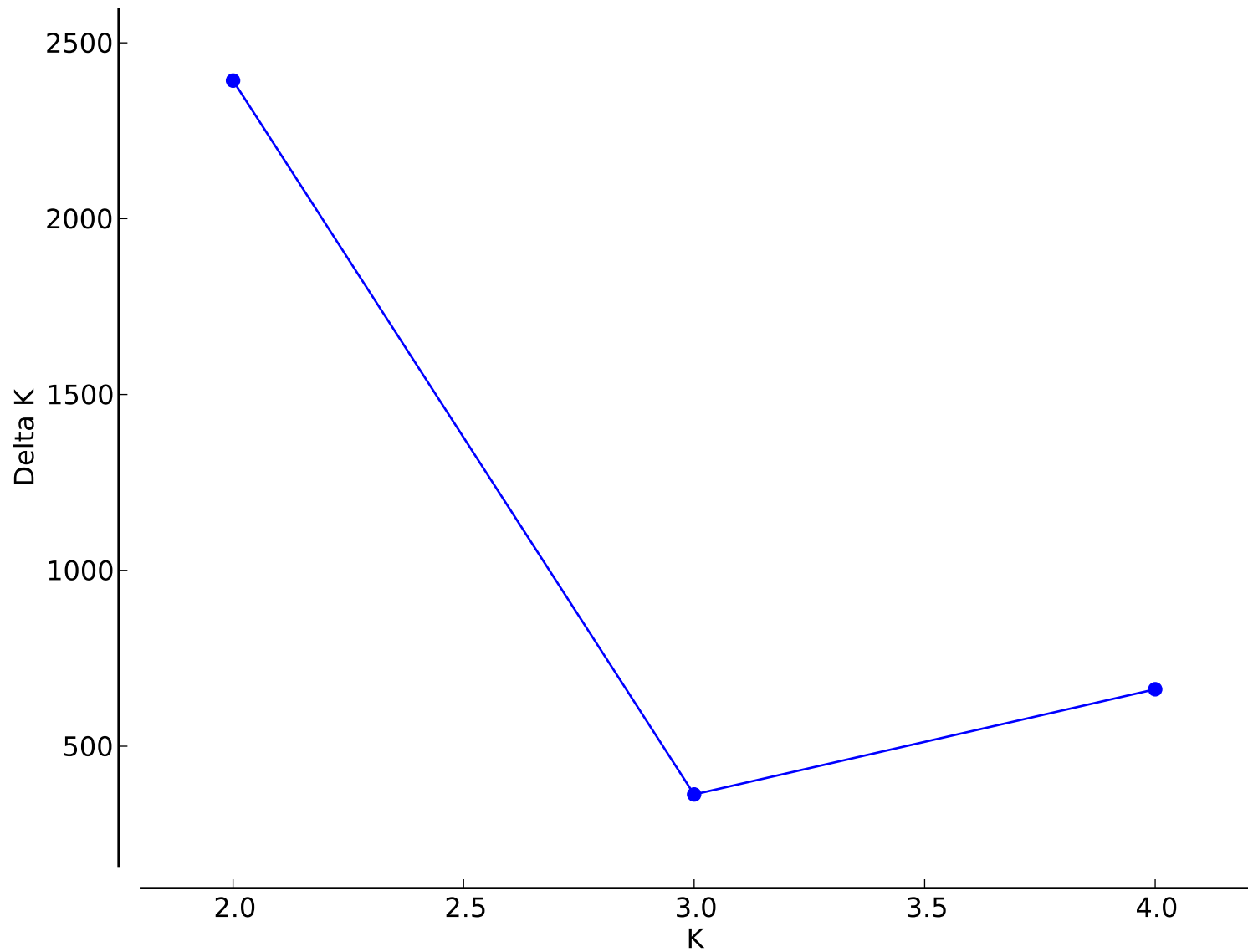

Supplement: S2 Fig — According to the ΔK, K = 2 represents the optimal structure partition in our dataset. (PDF) [file pone.0216903.s002.pdf]
