# Supplementary material for: Genetic and morphological identification of a recurrent Dicksonia tree fern hybrid in New Zealand
Source: PLoS One. 2019 May 20;14(5):e0216903. doi: 10.1371/journal.pone.0216903 (PMC6527230; doi:10.1371/journal.pone.0216903)
Supplement: S1 Table — (DOCX) [file pone.0216903.s003.docx]

Table S1. Newly developed microsatellite primers for New Zealand *Dicksonia*.

| Locus name | Primer sequences (5’-3’) | Allele size range (bp) | T_a_ (°C) | GenBank accession no. |
| --- | --- | --- | --- | --- |
| DicMic104 | F: CAACCCCGACATAGACACC  R: TGGAGGATAAGAGAGTGTGGAA | 114-121 | 55 | KY907147 |
| DicMic109 | F: CGGACGCCATGCCTTCAT  R: TTCCCTCGAATTCGCAACGT | 125-131 | 55 | KY907148 |
